# Response Inhibition and Interference Suppression in Individuals With Down Syndrome Compared to Typically Developing Children

**DOI:** 10.3389/fpsyg.2018.00660

**Published:** 2018-05-04

**Authors:** Laura Traverso, Martina Fontana, Maria Carmen Usai, Maria C. Passolunghi

**Affiliations:** ^1^Department of Education Sciences, University of Genoa, Genoa, Italy; ^2^Department of Life Sciences, University of Trieste, Trieste, Italy

**Keywords:** Down Syndrome, executive function, inhibition, interference suppression, response inhibition

## Abstract

The present study aims to investigate inhibition in individuals with Down Syndrome compared to typically developing children with different inhibitory tasks tapping response inhibition and interference suppression. Previous studies that aimed to investigate inhibition in individuals with Down Syndrome reported contradictory results that are difficult to compare given the different types of inhibitory tasks used and the lack of reference to a theoretical model of inhibition that was tested in children (see Bunge et al., 2002; Gandolfi et al., 2014). Three groups took part in the study: 32 individuals with Down Syndrome (DS) with a mean age of 14 years and 4 months, 35 typically developing children 5 years of age (5TD), and 30 typically developing children 6 years of age (6TD). No difference emerged among the groups in fluid intelligence. Based on a confirmatory factor analysis, two different inhibition factors were identified (response inhibition and interference suppression), and two composite scores were calculated. An ANOVA was then executed with the composite inhibitory scores as dependent variables and group membership as the between-subject variable to explore the group differences in inhibition components. The 6TD group outperformed the 5TD group in both response inhibition and interference suppression component scores. No differences were found in both inhibition components between the DS group and 5TD. In contrast, the 6TD group outperformed the DS group in both response inhibition and in the interference suppression component's scores. Summarizing, our findings show that both response inhibition and interference suppression significantly increased during school transition and that individuals with DS showed a delay in both response inhibition and interference suppression components compared to typically developing 6-year-olds, but their performance was similar to typically developing 5-year-olds.

## Introduction

Down Syndrome (DS) is the most common genetic syndrome associated with intellectual disability and affects ~1 in 700 newborns (Sherman et al., 2007; Mégarbané et al., 2009). Individuals with DS seem to have higher psychopathological risk than individuals with other intellectual disabilities (Gath and Gumley, 1986; Collacott et al., 1992; Dykens, 2007; Tassé et al., 2016). Therefore, acquiring more information on the weaknesses and strengths of the neuropsychological profile of individuals with DS is necessary for planning interventions.

Individuals with DS are usually characterized by moderate to severe learning disabilities and relative language impairments, with greater expressive difficulties than receptive ones (Fowler et al., 1994; Abbeduto et al., 2001; Laws and Bishop, 2004; Fidler and Nadel, 2007; Næss et al., 2011). Research on other cognitive abilities has focused mainly on memory resources, particularly working memory (Jarrold et al., 2000; Lanfranchi et al., 2004, 2012; Baddeley and Jarrold, 2007). People with DS have poorer working memory performance than controls, especially on tasks that require verbal processing compared to tasks with visual and spatial stimuli (Jarrold and Baddeley, 1997; Jarrold et al., 1999). This difference seems to be independent of the acoustic deficits typical of DS (Jarrold et al., 2000).

There is widespread agreement about impairments in executive function (Costanzo et al., 2013; Lee et al., 2015), a set of general-purpose control processes that regulate one's thoughts and behaviors (Miyake and Friedman, 2012). However, in the literature examining the cognitive profile of individuals with DS, there is a lack of information about inhibition, one of the core components of executive function (Miyake et al., 2000; Diamond, 2013). Inhibition has been considered to play a central role in cognitive development. Klenberg et al. (2001) claim that the development of basic inhibitory functions may precede the development of more complex cognitive functions. Miyake and Friedman (2012) speculate that inhibition may be a general resource for other executive functions. Because inhibition plays an important role in several cognitive activities, it is reasonable that an investigation into this ability may contribute to explaining cognitive impairments. Nevertheless, to date, only a few studies have examined the diverse inhibition components in individuals with DS, and the results are not consistent.

## Inhibition development

Inhibition processes generally refer to the ability to control one's mental processes and responses, to ignore an internal or external prompt and to perform an alternative action (Diamond, 2013). Studies that focus on inhibition have commonly described this ability as a multi-componential construct that includes different dimensions that are useful to perform different tasks (Dempster, 1993; Harnishfeger, 1995; Nigg, 2000; Diamond, 2013). For example, Diamond (2013) argues that inhibition comprises the ability to control irrelevant information at the level of thought and memories (cognitive inhibition), the ability to manage irrelevant data when acquiring information (inhibition at the level of attention), and the ability to control an action at the level of behavior (response inhibition). The concept of inhibition has been widely used and studied (i.e., Dempster and Brainerd, 1995). However, the psychometric construct of inhibition has been investigated only in recent decades (i.e., Friedman and Miyake, 2004). Using a latent variable approach, Rey-Mermet et al. (2017) demonstrated that a two-factor model in which two components, the inhibition of prepotent responses (the ability to suppress dominant responses) and the resistance to distracter interference (the ability to ignore distracting information or to suppress competing response tendencies), were distinguishable best explained the data observed in young and older adults (see also Stahl et al., 2014). However, this evidence collected with adults may not be applied to the early stages of development. As argued by Friedman and Miyake (2004) and observed by Bunge et al. (2002) in an fMRI study, children and adults may be characterized by different inhibition processes. Although a response inhibition component was not distinguishable in study by Friedman and Miyake (2004), in Bunge et al. (2002) study, different activation patterns for interference suppression and response inhibition were observed in children.

Recently, Gandolfi et al. (2014) proposed an empirical investigation of the latent organization of inhibitory processes in early childhood. They suggested that a unitary model was more useful for describing inhibitory processes in younger children (24- to 32-month-old children), whereas a two-factor model showed the best fit in children aged 36–48 months. Specifically, in 3- to 4-year-old children, Gandolfi et al. (2014) distinguished a response inhibition component from an interference suppression component (see also Bunge et al., 2002; Martin-Rhee and Bialystok, 2008; Cragg, 2016, in which interference at the level of response and interference at the level of the stimulus were considered, corresponding to what we define as the response inhibition and interference suppression components, respectively). The first component, “response inhibition,” significantly predicted the children's performance in tasks such as Go-No/Go, in which the child is presented with a stimulus that activates an automatic response that must be suppressed to give the correct response. The second component, “interference suppression,” explained performance in tasks such as the Flanker task, in which the child is presented with a stimulus that shows ambivalent data (the target and the flankers). In these tasks, the child must control the interference due to the stimulus characteristic and focus on the relevant information to give the correct response. This evidence may suggest that diverse inhibition components may emerge at different stages of development. For example, interference suppression may emerge after response inhibition, and it may be responsible for the differences between younger and older children in performing tasks in which interference must be controlled.

## Inhibition in down syndrome

Reviewing the literature of the last 20 years, to the best of our knowledge, we were able to identify 10 studies in which at least one inhibition task was proposed to a sample of individuals with DS (Table 1).

**Table 1:** Previous studies examining inhibition in individuals with Down Syndrome.

**Authors and aims**	**Sample**	**Investigated skills**	**Inhibition tasks**	**Results**
(Pennington et al., 2003). Evaluated hippocampal and prefrontal functions in individuals with DS.	*N* = 56 (28 DS; 28 TD) matched for MA. Age: DS average CA = 14.7 years; TD average CA = 4.9 years.	Inhibition; verbal and visual long-term memory; planning; fluency; spatial and verbal short-term memory.	Inhibition: Stopping task (Logan et al., 1984, 1997) Accuracy was recorded.	Inhibition: No significant difference between DS group and MA control group in stopping task. Effect size d_Cohen_ = −0.63.
(Rowe et al., 2006). Investigated EF in adults with DS.	*N* = 52 (26 DS; 26 LD). Age: DS range CA = 23–40 years; LD range CA = 19–55 years.	Inhibition/perseveration; set shifting; planning/problem solving; working memory; digit span; spatial span; fluency attention; verbal ability; motor speed.	Inhibition/perseveration: Finger tapping (Luria, 1980) Accuracy was recorded.	Inhibition: DS group scored at a lower level than the control group in finger tapping (*t* = 5.74, *p* = 0.20). After Bonferroni correction, the difference was no longer significant. Effect size d_Cohen_ = 0.93.
(Cornish et al., 2007). Study 2: Compared the trajectories of different aspects of attention (selective, sustained, inhibition) in three developmental disorders: FXS, DS, and WS.	*N* = 100 (25 DS; 25 FXS; 50 TD) matched for MA. Only boys. Age: DS average CA = 11.17 years and average MA = 6.09 years; FXS average CA = 10.88 years and average MA = 6.77 years; TD average CA = 7.78 years and average MA = 7.37 years.	Inhibition; selective and sustained attention.	Inhibition: Walk task-TEA-Ch (Manly et al., 1999) Accuracy was recorded.	Inhibition: No significant differences between DS and control group on Walk task accuracy (Bonferroni correction was used). Effect size d_Cohen_ = 0.17.
(Lanfranchi et al., 2010). Investigated performance on EF tasks on individuals with DS.	*N* = 30 (15 DS; 15 TD) matched for MA. Age: DS average CA = 15.2 years and average MA = 5.9 years; TD average CA = 5.9 years.	Inhibition; working memory; set shifting; conceptual shifting; planning; fluency; sustained attention	Inhibition:Day/Night Stroop Task (Gerstadt et al., 1994) Accuracy was recorded.	Inhibition: Significant difference between DS and normotypical control group in the experimental condition of Stroop task accuracy (*t* = −2.31, *p* = 0.028). Effect size d_Cohen_ = 0.87.
(Brunamonti et al., 2011). Evaluated the profile of cognitive control of movement in DS	*N* = 18 (9 DS; 9 LD) matched for MA. Age: DS average CA = 18.2 years; LD average CA = 15.3 years; MA = 9.0–9.6 years.	Inhibitory control; cognitive control of the movement.	Inhibitory control: Counterdemanding task (stop signal reaction time—SSRT) (Brunamonti et al., 2011) Reaction time was recorded.	Inhibition: Significantly longer reaction time (RT) in DS group compared to normotypical control group in the go process (average 570.9; SE 20.9; *t*-test; *p* < 0.01), no difference in stop processes. Effect size d_Cohen_ = −1.62; d_Cohen_ = −0.14.
(Borella et al., 2013). Investigated whether individuals with DS have a specific or general deficit in inhibitory abilities.	*N* = 38 (19 DS; 19 TD) children matched for MA. Age: DS average CA = 14.5 years and average MA = 5.6 years; TD average CA = 5.2 years.	Inhibition (Friedman and Miyake, 2004); working memory.	Prepotent response inhibition: Animal Stroop (adapted from Wright et al., 2003 by Nichelli et al. (2004)). Accuracy and reaction time were recorded. Resistance to proactive interference: Proactive interference (PI) task (adapted from Borella et al., 2010). Accuracy was recorded. Response to distracter inhibition: Directed forgetting—blocked method (adapted from Borella, 2006; Borella et al., 2011). Accuracy was recorded.	Prepotent response inhibition: No differences between the two groups in RT; participants with DS made more mistakes than TD (*F* = 6.64, *p* < 0.05). Effect size interference index accuracy d_Cohen_ = −0.86, response time d_Cohen =_ 0.06. Resistance to proactive interference: significant difference between DS and TD groups in resistance to proactive interference accuracy (*F* = 5.86, *p* < 0.05). Effect size intrusion errors d_Cohen_ = −0.81. Response to distracter inhibition: Individuals with DS performed worse than TD children considering accuracy, the word recalled in the second half of the list (*F* = 8.73, *p* < 0.05). Effect size first half d_Cohen_ = 0.43.
(Carney et al., 2013). Evaluated EF in DS and WS.	*N* = 75 (25 DS; 24 WS; 26 TD). Participants were not individually matched. Age: DS average CA = 10.4–18.11 years; WS average CA = 8.1–18.11 years; TD average CA = 5.0–8.0 years.	Inhibition; working memory; fluency; set shifting.	Inhibition: Verbal Inhibition, Motor Inhibition (VIMI) task (Henry et al., 2012) Accuracy and time were recorded.	Inhibition: No significant differences between DS and TD group. Effect size, verbal errors, d_Cohen_ = −0.40; verbal time d_Cohen_ = −0.06; visuospatial errors, d_Cohen_ = 1.19 visuospatial time, d_Cohen_ = 0.27.
(Costanzo et al., 2013). Evaluated the aetiological specificity hypotheses pertaining to EF by comparing individuals with intellectual disability of different etiology (DS and WS).	*N* = 46 (15 DS; 15 WS; 16 TD) matched for MA. Age: WS: average CA = 17.6 years and average MA = 6.7 years; DS: average CA = 14.5 years and average MA = 6.2 years; TD: average CA = 7.4 years and average MA = 6.9 years.	Response inhibition; attention; short-term and working memory; planning; categorization; shifting.	Verbal inhibition: Stroop task (Stroop, 1935) Time was considered. Visual inhibition: Go-No-Go (Van der Meere et al., 2005) Accuracy and time were recorded.	Verbal Inhibition: Significant difference in time emerged on Stroop task [*F*_(2, 46)_ = 7.27, *p* < 0.01]. Effect size d_Cohen_ = −1.17. Visual inhibition: no difference in the Go-No-Go task. Effect size accuracy d_Cohen_ = 0.87; response time d_Cohen_ = 0.76.
(Schott and Holfelder, 2015). Examined motor skills and EF as well as the relationship between these two performance domains.	*N* = 36 (18 DS; 18 TD) matched for sex and age. Age: DS: average CA = 9.06 years; TD: average CA = 8.99 years.	Inhibitory control; motor assessment; executive function; set switching.	Response suppression and distraction: Trail-Making Test for young children (Trails-P) Accuracy and time and a composite score were considered.	Inhibition: Significant differences between DS and TD groups considering number of errors, mean time and efficiency (*p* < 0.001). Effect size for response suppression accuracy d_Cohen_ = −1.82; response time d_Cohen_ = −2.05.
(Amadó et al., 2016) Investigated the links between EF and social cognition among children with DS.	*N* = 90 (30 DS; 60 TD). Age: DS: average CA = 8.54 years TD matched: 30 for MA and 30 for LD.	Inhibition; working memory; and cognitive flexibility (EF, Miyake et al., 2000); social cognition.	Inhibition: Day-Night Stroop task (Gerstadt et al., 1994) Accuracy was recorded.	Inhibition: DS group underperformed both CA (*p* < 0.001) and LD groups (*p* < 0.01). Effect size d_Cohen_ = 1.38.

*d_Cohen_ with pooled standard deviation*.

Although the study designs were comparable, contradictory findings emerged. In some studies, the DS group performed significantly worse on the inhibitory task administered compared to the control group (Lanfranchi et al., 2010; Schott and Holfelder, 2015; Amadó et al., 2016). In other studies, no difference emerged (Pennington et al., 2003; Cornish et al., 2007; Carney et al., 2013). Finally, in some studies, mixed results were reported (Rowe et al., 2006; Brunamonti et al., 2011; Borella et al., 2013; Costanzo et al., 2013). For example, Borella et al. (2013) found a significant difference in accuracy on all three tasks, although no difference emerged in response time in one of the tasks. In Costanzo et al. (2013), a difference was found for the Stroop task but not for the Go-No/Go task.

These inconsistencies seem to highlight the need to differentiate performance across inhibition components rather than by considering a unitary inhibition dimension. Nevertheless, comparing these results to derive conclusions about the development of the inhibition component in DS is not easy. In most studies, only one task was used. Therefore, contradictory findings may be due to the differences in the tasks used. For example, in both Amadó et al. (2016) and Lanfranchi et al. (2010), accuracy in a Day-Night Stroop task was considered, and in both studies, a significant difference between the DS and the control group was reported. However, these consistent results may involve non-inhibition abilities necessary to perform the task or diverse inhibition components required by the Stroop task that are not assessed with other inhibition tasks. Conversely, in Costanzo et al. (2013) and Borella et al. (2013), a Stroop task was used, and these two studies reported different results using response time and accuracy as indicators. In Costanzo et al. (2013), the DS sample differed from the control group in response time but not in accuracy, whereas the opposite pattern was observed in Borella et al. (2013). As reported by Friedman and Miyake (2004), several problems arise when single and raw inhibition scores are considered. Moreover, although these studies provide useful information about diverse cognitive abilities in DS individuals, the fact that only one task was used to assess inhibition does not allow us to investigate the development of the diverse inhibition components. Only the study by Borella et al. (2013) used three inhibition tasks to assess the three inhibition components initially hypothesized for adults by Friedman and Miyake (2004). In the other studies, the proposed tasks are generally defined as inhibition tasks without providing clarification of the specific component that may be assessed with each task. If we consider the model proposed and verified for children (see Bunge et al., 2002; Gandolfi et al., 2014) in which response inhibition and interference suppression were identified, previous studies on individuals with DS have mostly investigated response inhibition (see inhibition task column in Table 1, in which diverse response inhibition tasks were included, such as the Go-No/Go task, the Finger Tapping task, and the Stroop task) rather than the interference suppression component of inhibition. In summary, there is a need for a study that analyses the development of response inhibition and interference suppression components (following the two-factor model proposed and tested with children by Gandolfi et al., 2014) in a DS sample.

## The present study

The current study aims to investigate diverse inhibition components in typically developing children and individuals with DS. In agreement with several authors (Friedman and Miyake, 2004; Diamond, 2013), we consider inhibition as having a multicomponent nature, and we hypothesize that at least these two components will be identifiable at this stage of development in TD children (Gandolfi et al., 2014). Specifically, we aim to verify whether two inhibition components, response inhibition and interference suppression, can be found in typically developing children at five (5TD) and 6 years of age (6TD). In addition, considering that inhibition abilities undergo rapid changes in the typical population at the ages considered (Davidson et al., 2006), we investigate whether differences in response inhibition and interference suppression efficiency may be found between TD children aged 5 and 6 years. Moreover, response inhibition and interference suppression are examined in individuals with DS with the same mental age of the two TD groups. Our aims are to investigate whether the DS and the TD groups differ in inhibition performance and to acquire more information concerning inhibition development in DS by comparing this group with two TD groups that may differ in the level of inhibition development.

In contrast to previous studies in which only single task scores were considered, we aimed to at least partially overcome the problems due to task impurity (see Friedman and Miyake, 2004) by creating a composite score for each inhibition component. The difference between typical children of 5 and 6 years and individuals with DS matched for mental age is examined with consideration of these composite scores. Borella et al. (2013) reported general impairment in the diverse inhibition components investigated; thus, we may hypothesize that significant differences will emerge in both components. However, Borella et al. (2013) refer to an adult model of inhibition, whereas we aim to investigate for the first time two inhibition components that have been identified in typical children in a sample of youth with DS.

## Methods

### Participants

A final sample of 97 individuals belonging to three groups took part in this study. Thirty-two individuals with Down Syndrome (DS), 22 girls and 10 boys with a mean age of 14 years and 4 months (*M*_age_ 173.75 in months, *S.D*. 65.17, range: 73–299 months), were included in the DS group. Thirty-five typically developing children, 18 girls and 17 boys with a mean age of 5 years and 6 months (*M*_age_ 67.37 in months, *S.D*. 2.85, range: 62–71 months), were included in the typically developing control group of 5-year-olds (5TD). Thirty typically developing children, 13 girls and 17 boys with a mean age of 6 years and 2 months (*M*_age_ 74.40 in months, *S.D*. 4.42, range: 72–84 months), were included in the typically developing control group of 6-year-olds (6TD). Individuals with DS had trisomy 21 without mosaicism and were recruited from two treatment centers in the north of Italy. Typically developing children were recruited from different educational services in the same area. None of the children had a history of neurological impairment or developmental disabilities.

### Procedure

A battery of inhibition tasks was administered to the three groups by trained psychologists. All participants were tested individually in a quiet room in two separate testing sessions, each lasting ~20–30 min, at an interval of 3–4 days. The DS group was assessed in the treatment center, and the TD children were tested at educational services. The families were previously informed about the aims of the study and about the activities in which the participants were involved. A written informed consent form was completed by the parents before testing began.

All tasks consisted of well-known inhibition paradigms. These tasks have been widely used with children and did not show any floor or ceiling effect in the mental age range of interest (Davidson et al., 2006; Traverso et al., 2015). These tasks minimize the non-executive function abilities required. Basic knowledge (such as colors) and simple responses (such as pointing or pressing) are required to perform the tasks. Finally, all tasks (except for the Go/No-Go) included practice trials before the test began. The examiner gave the instructions and then conducted the practice trials to verify whether the child had comprehended the requirements of the task.

### Measures

The Colored Progressive Matrices Test (Raven, 1947; Belacchi et al., 2008) was administered to measure fluid intelligence and was used as a screening measure to match fluid intelligence between the DS group and the two TD groups. It is a multiple-choice test of abstract reasoning in which the child is required to complete a geometrical figure by choosing the missing piece among six possible drawings. The tasks included 36 items. The items varied in difficulty. The score was the number of correct responses (CPM, expected range 0–36).

#### Inhibition battery

To assess inhibition, the following tasks were administered.

##### Go/no-go task (adapted from Berlin and Bohlin, 2002)

The Go/No-Go task is a well-known paradigm that tests the abilities of both adults and children to inhibit prepotent responses (Durston et al., 2002; Verbruggen and Logan, 2008). The children were asked to restrain an automatic response. While in front of a computer screen, the child was instructed to press the space bar according to the instructions given by the examiner for the following condition: “Press the space bar when you see a blue figure; do not press when you see a red figure” (24 blue items and six red items). The percentage of go responses was 80%. The stimulus duration was 3,000 ms, and the blank page that appeared after each stimulus lasted 1,000 ms. The sum of the correct responses in the no-go condition was recorded (Go/No-Go Accuracy, expected range 0–6). Test-retest reliability (Pearson's r) was calculated in a sample of 75 typically developing children (age range 62–76 months, *M*_age_ = 68.64; *S.D*. = 3.5) was 0.55, *p* < 0.0005 (unpublished results from the data set used in Traverso et al., 2015). Cronbach's alphas calculated in the present study were 0.71 in the TD group and 0.83 in the DS group.

##### Preschool matching familiar figure task (PMFFT, adapted by Kagan, 1966; Traverso et al., 2016)

This task measures the child's ability to restrain impulsive responses and to compare the target with all of the pictures by shifting attention from the target to each alternative. The children were asked to perform 14 trials, selecting among five different alternatives the figure that was identical to the target picture at the top of the page. The number of errors (PMFFT Errors, expected range 0–56) and the mean latency between the presentation of the item and the child's response (PMFFT Time, expected range 0-no limit) were recorded. Cronbach's alphas calculated in a sample of 174 children (*M*_age_ = 60.04) were 0.67 for PMFFT Errors and 0.95 for PMFFT Time (Traverso et al., 2016). Cronbach's alpha calculated in the present study for PMFFT Accuracy was 0.76 in the TD group and 0.85 in the DS group. Cronbach's alpha for PMFFT Time was 0.94 for both groups.

##### Fish flanker task (adapted from Ridderinkhof and van der Molen, 1995; Gandolfi et al., 2014; Traverso et al., 2015)

The Flanker task is a well-known paradigm that is used to evaluate the ability to inhibit irrelevant interfering stimuli (Eriksen and Eriksen, 1974; Kramer et al., 1994). The children were required to respond to a left or right fish presented at the center of the computer screen by pressing a left or right response button. The fish was flanked by two fishes pointing in the same direction (congruent condition, 16 items) or in the opposite direction (incongruent condition, 16 items). After a brief training consisting of four items (two of each condition), 48 items were randomly presented (16 items per condition, half left and half right). A warning cross (500 ms in duration) preceded the stimulus. After the response, the screen turned blank for 500 ms. Accuracies (Flanker Accuracy, expected range 0–16) and response times (Flanker Time) in the incongruent condition were recorded. Test-retest reliability (Pearson's r) calculated in a sample of 43 typically developing children (age range 62–75 months, *M*_age_ = 68.60; *S.D*. = 3.5) was 0.42, *p* = 0.002 and 0.56, *p* < 0.001 for Flanker Accuracy and Flanker Time, respectively (Usai et al., 2017). Cronbach's alphas calculated in the present study for Flanker Accuracy were 0.96 in the TD group and 0.81 in the DS group. Cronbach's alphas for Flanker Time were 0.96 in the TD group and 0.93 in the DS group.

##### Dots task (adapted by Diamond et al., 2007; Traverso et al., 2015)

This task is a high cognitive conflict task (see Diamond et al., 2007; Diamond and Lee, 2011). A heart or a flower appears on the right or left of a computer screen. The child is told that he must press on the same side of the heart but on the opposite side of the flower, which requires inhibiting the tendency to respond on the side where the stimulus appeared and to control the response based on which stimulus appears. After a brief training session with heart and flower items, the test began, and hearts and flowers were intermixed in the test. The sum of correct responses (Dots Accuracy, expected range 0–20) and the response time (Dots Time) were recorded for each child. Test-retest reliability (Pearson's r) calculated in a sample of 43 typically developing children (age range 62–75 months, *M*_age_ = 68.60; *S.D*. = 3.5) was 0.62 (*p* < 0.001) for Dots Accuracy and 0.72 (*p* > 0. 001) for Dots Time (Usai et al., 2017). Cronbach's alpha calculated in the present study for Dots Accuracy was 0.97 in the TD group and 0.80 in the DS group. Cronbach's alpha for Dots Time was 0.89 in the TD group and 0.85 in the DS group.

### Statistical analyses

Descriptive analyses and ANOVAs on CPM and inhibitory measures were conducted to compare the three groups' performance considering both accuracy and response time scores. The relation between accuracy and response time was investigated with bivariate correlations. A confirmatory factor analysis (CFA) was performed using the TD group's inhibitory task scores to verify the characteristics of the inhibition construct in early childhood. Multiple fit indices were considered to compare models (for an extensive description, see, e.g., Schermelleh-Engel et al., 2003): the X^2^ statistic, the Comparative Fit Index (CFI), the root mean square error of approximation (RMSEA), the standardized root mean squared residual (SRMR), the Akaike Information Criterion (AIC), and the Bayesian information criterion (BIC). The X^2^ test was used to evaluate the appropriateness of the CFA model. Non-significant X^2^ values indicated a minor difference between the covariance matrix generated by the model and the observed matrix and thus an acceptable fit. CFI values > 0.97 are indicative of a good fit, whereas values > 0.95 may be interpreted as an acceptable fit (Schermelleh-Engel et al., 2003). RMSEA values ≤ 0.05 represent a good fit, values between 0.05 and 0.08 represent an adequate fit, values between 0.08 and 0.10 represent a mediocre fit, and values > 0.10 are not acceptable (Browne and Cudeck, 1993). The SRMR is the square root of the averaged squared residuals (i.e., the differences between the observed and predicted co-variances). SRMR values < 0.10 are acceptable; however, values lower than 0.05 represent a good fit (Schermelleh-Engel et al., 2003). Based on the CFA results, composite scores were calculated as the mean of the inhibitory z-score to represent the latent inhibitory dimensions. Finally, an ANOVA was conducted with the composite inhibitory scores as dependent variables and group membership as the between-subject variable to explore group differences in the inhibition components.

## Results

Descriptive statistics and ANOVA results for the three groups are shown in Table 2. A univariate analysis of variance showed no significant difference in the CPM score. In contrast, significant differences among the groups were found for all the inhibition tasks with the exception of the Dots Time score.

**Table 2:** Descriptive statistics of measures for the three groups and results of the comparisons among groups (ANOVA) for CPM and inhibition tasks.

	**Groups**	**Mean**	***S.D*.**	**Min**	**Max**	***F***	**Sig**.	**Comparisons**	**Effect size**
CPM	5TD	18.43	2.13	16	24	2.306	0.105	5TD = 6TD	0.69
	6TD	20.33	3.21	16	27			DS = 5TD	0.31
	DS	19.63	5.03	13	31			DS = 6TD	0.16
PMFFT	5TD	13.49	5.95	0.00	26.00	8.41	0.0001	5TD > 6TD^**^	1.01
Errors									
	6TD	8.10	3.99	0.00	16.00			DS = 5TD	0.12
	DS	14.38	8.65	2.00	43.00			DS < 6TD^**^	0.89
PMFFT	TD5	6.79	5.20	1.93	26.91	8.31	0.0001	6TD = 5TD	0.70
Time									
	6TD	10.35	4.52	3.86	24.77			DS > 5TD^***^	0.87
	DS	14.27	10.84	5.40	57.26			DS = 6TD	0.45
Go/No-Go	5TD	5.06	1.39	0.00	6.00	5.06	0.008	5TD = 6TD	0.23
Raw Score									
	6TD	5.37	1.22	0.00	6.00			DS = 5TD	0.52
	DS	4.13	2.09	0.00	6.00			DS < 6TD^*^	0.70
Go/No-Go	5TD	246.60	152.22	1.00	403.43	2.53	0.085	5TD = 6TD	0.30
Transformed	6TD	292.23	142.43	1.00	403.43			DS = 5TD	0.27
	DS	199.76	186.72	1.00	403.43			DS = 6TD	0.54
Flanker Accuracy	5TD	8.74	4.45	0.00	15.00	6.08	0.003	5TD < 6TD^**^	0.82
	6TD	12.42	4.14	1.00	16.00			DS = 5TD	0.35
	DS	10.30	4.08	2.00	16.00			DS = 6TD	0.50
Flanker	5TD	887.26	149.53	513.20	1146.10	14.91	0.0001	5TD = 6TD	0.68
Time									
	6TD	1058.15	322.12	417.60	1932.40			DS > 5TD^***^	0.99
	DS	3230.11	3332.41	537.69	13822.30			DS > 6TD^***^	0.87
Dots	5TD	12.83	3.76	4.00	19.00	13.07	0.0001	5TD < 6TD^*^	0.50
Accuracy									
	6TD	14.80	3.80	8.00	20.00			DS < 5TD^*^	0.74
	DS	10.63	1.54	8.00	15.00			DS < 6TD^***^	1.41
Dots	5TD	1270.70	378.05	570.00	2055.20	2.37	0.099	5TD = 6TD	0.23
Time	6TD	1367.97	439.20	547.70	2254.10			DS = 5TD	0.43
	DS	1718.34	1410.30	244.00	5968.30			DS = 6TD	0.32

* p < 0.05;

** p < 0.001;

*** *p < 0.0001. Time is reported in seconds for the Preschool Matching Familiar Figure Task Time (PMFFT Time) and in milliseconds for the Flanker (Flanker Time) and Dots tasks (Dots Time)*.

*Post-hoc* tests using Bonferroni correction revealed that 6-year-olds outperformed 5-year-olds in PMFFT Errors (6TD made fewer errors than 5TD), Flanker Accuracy and Dots Accuracy. The DS group showed high variability in all tasks. This group performed worse than the 6TD group but was similar to the 5TD group in PMFFT accuracy. The opposite was observed for PMFFT time, and the DS group showed a similar response time to the 6TD and a higher response time than 5TD. A significant difference emerged in the Go/No-Go task between the 6TD and DS groups; however, this difference disappeared when a mathematical transformation (exponential function, Kline, 2005) was applied to the Go/No-Go raw score to obtain acceptable skewness and kurtosis parameters. For Flanker Accuracy, the DS group showed similar accuracy scores to the two TD groups and a higher response time than both the 5TD and the 6TD groups. Finally, the DS group showed worse performance in Dots Accuracy than 5TD and 6TD, and no differences emerged in Dots Time.

Zero-order correlations among tasks are reported for the two TD groups (Table 3) and the DS group (Table 4).

**Table 3:** Zero-order correlation through inhibition tasks, CPM and age (in months) in the 5TD group (upper triangle) and in the 6TD group (lower triangle).

	**1**	**2**	**3**	**4**	**5**	**6**	**7**	**8**	**9**	**10**
PMFFT errors	1	−0.592^**^	−0.589^**^	−0.374^*^	0.156	−0.166	−0.041	−0.096	−0.052	0.133
PMFFT time	−0.543^**^	1	0.232	0.199	−0.148	0.154	−0.108	0.121	0.134	−0.341^*^
Go/No-Go raw score	−0.348	0.169	1	0.807^**^	−0.036	0.264	0.154	0.249	0.081	−0.213
Go/No-Go transformed	−0.198	0.054	0.796^**^	1	0.008	0.211	0.231	0.23	−0.069	−0.179
Flanker accuracy	−0.247	0.463^*^	0.14	0.116	1	−0.347^*^	0.287	−0.047	0.216	0.11
Flanker time	−0.297	0.409^*^	0.191	0.076	0.391^*^	1	0.056	0.491^**^	0.113	−0.059
Dots accuracy	−0.319	0.394^*^	0.367^*^	0.34	0.463^**^	0.347	1	0.557^**^	0.057	−0.049
Dots time	−0.315	292	0.414^*^	0.414^*^	0.408^*^	0.596^*^	0.754^**^	1	0.153	−0.253
CPM	−0.301	0.396^*^	0.065	−0.037	0.513^**^	0.436^*^	0.124	0.322	1	0.171
Age	−0.11	−0.073	0.087	0.053	0.269	0.023	−0.005	−0.087	0.36	1

* p < 0.05;

** *p < 0.001*.

**Table 4:** Zero-order correlation through inhibitory tasks, CPM and age (in months) in the DS group.

	**1**	**2**	**3**	**4**	**5**	**6**	**7**	**8**	**9**	**10**
PMFFT errors	1	0.051	−0.360^*^	−0.348	−0.540^**^	0.184	−0.154	0.146	−0.583^**^	−0.267
PMFFT time		1	0.189	0.246	−0.055	0.295	−0.061	0.455^**^	−0.006	0.147
Go/No-Go raw score			1	0.893^**^	0.463^**^	−0.161	0.105	0.206	0.139	0.244
Go/No-Go transformed				1	0.413^*^	−0.16	0.046	0.155	0.105	0.247
Flanker accuracy					1	−0.246	0.243	−0.007	0.234	0.185
Flanker time						1	0.072	0.575^**^	−0.189	0.128
Dots accuracy							1	0.372^*^	0.173	0.113
Dots time								1	−0.084	0.02
CPM									1	0.071
Age										1

* p < 0.05;

** *p < 0.001*.

As expected, the inhibition task scores were not highly related (Willoughby et al., 2015). In the 5TD group, a significant association emerged between performance in the PMFFT (Errors) and the Go/No-Go tasks. In the 6TD group, the Dots Accuracy was positively correlated with the Flanker Accuracy, and the Dots Accuracy was related to the Go/No-Go performance. In the DS group, performance in the PMFFT (Errors) and the Go/No-Go tasks were associated, and the Flanker Accuracy was related to both the PMFFT (Errors) and the Go/No-Go Accuracy. Accuracy and response time correlated significantly in both the 5-year-old (*r* ranged from 0.347 to 0.592) and the 6-year-old (*r* ranged from 0.391 to 0.754) groups. However, in the DS group, only the Dots Accuracy and the Dots Time scores were related (*r* = 0.372). The CPM performance was associated with the PMFFT Time and the Flanker task (Time and Accuracy) in the 6TD group, no significant association emerged considering the 5TD group, and CPM was related to the PMFFT Time in the DS group. Finally, age was significantly related only to the PMFFT time in the 5TD group.

### Identifying the inhibitory components

**Figure 1:**
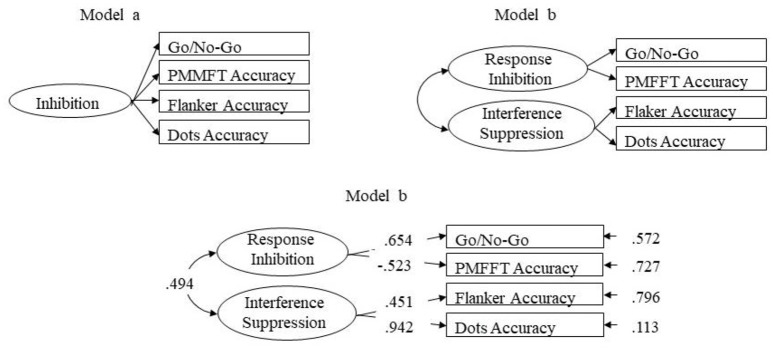
Inhibition models. Model b is the endorsed model (standardized parameters are reported).

To verify whether the two-factor model, in which response inhibition and interference suppression were distinguished, would be more useful to explain the observed data than a one-factor model (Figure 1), a series of CFAs based on raw data were performed using Mplus software (version 7.4) (Muthén and Muthén, 2007).

The unitary model had mediocre or unacceptable fit indices: χ^2^ = 5.014 *p* = 0.082, CFI = 0.872, SRMR = 0.060, RMSEA = 0.152, and 90% CI = [0.000, 0.325]. The two-factor model (Figure 1) showed the best fit: χ^2^ = 0.556 *p* = 0.456, CFI = 1.000, SRMR = 0.018, RMSEA = 0.000 and 90% CI = [0.000, 0.295]. All the factor loadings were significant (*t* values > 2).

### Investigating the inhibitory difference in DS and TD groups

Two composite scores representing response inhibition and interference suppression were calculated as the mean of the z-scores as follows: the z-score average of PMFFT Errors and Go/No-Go task Accuracy for response inhibition and the z-score average of Flanker Accuracy and Dots Accuracy for interference suppression (Table 5). These composite measures can be considered formative indicators of the two inhibitory factors found with the previous EFA (Willoughby et al., 2015). The results of an ANOVA conducted with the two composite inhibitory measures as dependent variables and group membership as the between-subjects variable showed that the three groups differed in both response inhibition, *F*_(2, 96)_ = 8.363 *p* < 0.001, and interference suppression, *F*_(2, 96)_ = 10.530 *p* < 0.001. The 6TD group outperformed the 5TD group in both the response inhibition (*p* = 0.008, d_Cohen_ = 0.94) and interference suppression (*p* = 0.001, d_Cohen_ = 0.83) components. No differences were found in either inhibition component between the SD and 5TD groups. In contrast, the 6TD group outperformed the DS group on the response inhibition component score (*p* = 0.001, d_Cohen_ = 0.96) and the interference suppression component score (*p* < 0.001, d_Cohen_ = 1.15).

**Table 5:** Descriptive statistics of inhibitory components in the three groups.

**Groups**	**Response inhibition**				**Interference suppression**			
	**Mean**	***S.D*.**	**Min**	**Max**	**Mean**	***S.D*.**	**Min**	**Max**
5TD	−0.192	1.11	−2.73	1.78	−0.169	0.820	−2.376	1.278
6TD	0.717	0.782	−1.30	2.21	0.517	0.849	−1.430	1.642
DS	−0.462	1.53	−5.14	1.93	−0.301	0.551	−1.309	0.947

## Discussion

The main goal of this study was to investigate diverse inhibition components in children and youth with DS compared to two groups of typically developing children aged 5 and 6 years matched for mental age. Specifically, we aimed to focus on response inhibition and on interference suppression components (see Bunge et al., 2002; Gandolfi et al., 2014). In contrast to previous studies in which only single task scores were examined, we considered both raw scores and composite scores as formative indicators of these two components, referring to a theoretical model of inhibition that was tested in children (Gandolfi et al., 2014).

### Inhibition in children with typical development

First, the performance of the two typically developing groups was analyzed. Although inhibition development has been widely documented and investigated in childhood in preschool more than in the transition to school (Carlson, 2005; Romine and Reynolds, 2005; Davidson et al., 2006; Garon et al., 2008), the developmental trajectories of this ability and its components are not yet clear. To acquire more information on atypical development, we argue that it is important to focus on inhibition changes in typically developing children.

Concerning single tasks, our results showed that children 6 years of age were more accurate than 5-year-olds in most of the tasks, although they did not have significant differences in general cognitive functioning measured with CPM. These findings are consistent with previous studies that documented a rapid improvement in accuracy on similar tasks in this age range (Davidson et al., 2006; Traverso et al., 2016). Moreover, the older children significantly increased their response time in the Preschool Matching Familiar Figure Task. In all three tasks in which response time was registered, it was significantly positively related (higher the time, greater the accuracy) to accuracy in both the 6-year-olds and the 5-year-olds. In middle childhood and adulthood, low response time is considered an index of a high level of inhibition. In contrast, Gerstadt et al. (1994) showed that in early childhood, children who took longer to respond were more likely to be correct. Diamond et al. (2002) demonstrated that it is possible to increase accuracy by encouraging children to wait before answering in a Stroop task, and some authors argue that the time is useful because it permits the dissipation of the prepotent response in children (Simpson et al., 2012; Ling et al., 2016). In an investigation of the performance of 3- to 6-year-old children on the Preschool Matching Familiar Task, in which no instruction to wait before answering was given, Traverso et al. (2016) observed that response time and accuracy were not related until the age of four and a half years. These results suggest that the interpretation of the time response may depend on age, accuracy, and task; consequently, it may not be a valid index of cognitive efficiency when these other parameters are not considered, at least in childhood (see Davidson et al., 2006; but see studies, i.e. Tamm et al., 2012), in which an application of ex Gaussian distribution to response time allowed the achievement of more fine-grained analyses of the distribution and consequently obtained much more information on cognitive profile than using raw response time, which was characterized by high variability and was not normally distributed.

As expected, the inhibition tasks did not correlate with each other (Willoughby et al., 2015; Rey-Mermet et al., 2017) in all three groups. Nevertheless, according to previous studies (see Gandolfi et al., 2014), the CFA demonstrated that a two-factor model in which response inhibition (Go/No-Go task and Preschool Matching Familiar Figure Task indicators) and interference suppression (Flanker Accuracy and Dots Accuracy indicators) were distinguishable best explained the data observed. In the Go/No-Go task and the Preschool Matching Familiar Figure Task, the child is required to focus on one attribute of the stimulus. In the Go/No-Go task, the child must look at the color of the figure and be able to control the response to press the spacebar. In the Preschool Matching Familiar Figure Task, the child must be able to consider the target and then the figure before pointing with the finger. In both tasks, the child is required to press/point or not to press/point according to the stimulus presented. Given the large majority of go stimuli and the diverse figures that need to be compared in the Preschool Matching Familiar Figure Task, in these tasks, the child usually must stop an automatic response or an impulsive tendency. In contrast, in both the Flanker Task and the Dots Task, the child must always give a response (press a computer key). Nevertheless, the child must analyse the type of stimulus that is presented to evaluate what type of response is correct. The stimuli presented are particularly challenging. In the Flanker Task, the child must be able to focus on the central fish; in the Dots Task, the child must focus on the type and side of the stimulus. Whereas, in the first type of tasks the child must decide to respond or not consider the stimulus, in the latter tasks, the child must choose between to different responses by managing the complexity of the stimulus. In these tasks, the child must suppress distracting information as well as competing response tendencies. Following the CFA, two composite scores were calculated as a formative index of response inhibition and interference suppression components. As suggested by Willoughby et al. (2015), formative indices may be a useful method to investigate EF development. However, it must be noted that this conceptual framing is consistent with the characterization of EF as a latent variable that is defined by (rather than giving rise to) individual performance across a set of performance-based tasks. Our results show that older children obtained higher scores than younger children in both response inhibition and interference suppression. These results may suggest that from 5 to 6 years of age, children increase both their ability to control an automatic response and their ability to manage interference. Previous studies have shown that performance on response inhibition tasks such as the Go/No-Go task undergoes significant changes in middle childhood (Brocki and Bohlin, 2004; Cragg and Nation, 2008). Similarly, an increase in performance on tasks that are supposed to require interference suppression was previously observed in middle childhood studies (Hommel et al., 2004). Both components improve during school transition, although Gandolfi et al. (2014) suggested that interference suppression emerges after response inhibition in pre-schoolers, and Cragg (2016) claimed that the improvements in performance on inhibition tasks in middle childhood may be due to development in what we define as interference suppression rather than response inhibition.

### Inhibition in individuals with down syndrome

With regard to task accuracy, the DS group showed worse performance than the 6-year-olds on the Preschool Matching Familiar Figure Task and worse performance than both groups in the Dots task. No differences were observed in the Go/No-Go task transformed variable (although a difference emerged in the raw score) and in the Flanker Task accuracy. Moreover, the DS group had a higher response time than the 5-year-olds on the Preschool Matching Familiar Figure Task and a higher response time than both control groups on the Flanker task. This inconsistent pattern is in line with the inhibition literature (Rey-Mermet et al., 2017) and with studies that have found high variability on cognitive tasks in the atypical development population (i.e., Tamm et al., 2012; van Belle et al., 2015). With reference to previous studies, as in Costanzo et al. (2013), no differences were observed in the Go/No-Go task, whereas a significant difference emerged in other tasks requiring response inhibition (although in tasks different from the tasks we used; see Lanfranchi et al., 2010; Schott and Holfelder, 2015; Amadó et al., 2016). For interference suppression tasks, to our knowledge, only a study by Merrill and O'dekirk (1994) used a Flanker paradigm, and individuals with DS showed more interference caused by the flankers (and higher response time) than controls. Otherwise, no difference emerged in our study.

One possible explanation for these mixed results may involve the non-executive abilities required by the task. In the Merrill and O'dekirk study, the flankers were letters; therefore, we cannot exclude the possibility that their results were due to the DS group's difficulties in verbal elaboration. Costanzo et al. (2013) explained their mixed results by arguing that the differences were due to the visual vs. verbal stimuli. However, in our study, the DS group performed worse on tasks in which visual stimuli must be processed (i.e., Preschool Matching Familiar Figure Task). In the Flanker Task, in contrast to the other tasks, the examiner used a brief story-telling paradigm to explain what the child was expected to do. Thus, it is possible that the children were more motivated to perform the Flanker task than the other tasks and that they were helped by a practical story rather than arbitrary and abstract rules for the task. Another possible explanation involves the difference in other executive demands of the task. For instance, the Dots task and the Preschool Matching Familiar Figure Task may require higher working memory than the other two tasks. Nevertheless, according to Munakata et al. (2011), the child needs to actively maintain the goal of the task in working memory in all types of inhibition tasks.

To discuss these mixed results, it is helpful to reflect on which variable was considered (accuracy vs. response time). Previous studies considered both accuracy and response time, and, as in our study, mixed results were reported. Nevertheless, it must be noted that in our study, accuracy was unrelated to response time in both the Flanker task and the Preschool Matching Familiar Task. This evidence may suggest that as early pre-schoolers (Traverso et al., 2016), individuals with DS are not able to control response time to be more accurate; thus, response time may not be a useful index of executive control in this population.

We speculate that focusing on single task differences makes it difficult to investigate the efficacy of the inhibition components (see Miyake et al., 2000; Willoughby et al., 2015). Consequently, we prefer to focus on inhibition composite scores as indices of response inhibition and interference suppression. When composite scores were considered, the DS group performed similarly to the younger children using both components. In contrast, a significant difference emerged between the older children and the DS group in both components. These results suggest that individuals with DS show a deficit in both response inhibition and interference suppression components when compared with a TD population that shows more mature inhibition abilities than the younger group of TD children. In previous studies, most of the tasks used required response inhibition. Our studies on the response inhibition component confirmed the evidence provided by Amadó et al. (2016), Lanfranchi et al. (2010), and Schott and Holfelder (2015). However, few studies have examined the interference suppression component. Moreover, to the best of our knowledge, this is the first study in which individuals with DS were compared with two typically developing groups at different stages of development.

In summary, our findings demonstrate that individuals with DS show a delay in inhibition development, but their performance is similar to the typical development of 5-year-old children. This evidence is consistent with the study by Borella et al. (2013), in which individuals with DS showed difficulties in tasks assessing diverse inhibition components. Moreover, it should be noted that even though differences emerged between the groups, the three groups had the same level of general cognitive functioning. These results suggest that significant differences in inhibition abilities may characterize groups with similar levels of general cognitive functioning in typical development. Consequently, when differences in individuals with DS and typically developing children are investigated, it is possible that mixed results will emerge due to the age of typically developing children with similar cognitive functioning, which may be characterized by diverse levels of inhibition development.

## Conclusion

To the best of our knowledge, in the last 20 years, only ten studies have examined the inhibition abilities of individuals with DS. These studies reported contradictory results and generally used only response inhibition tasks without referring to a theoretical model of inhibition (see Borella et al., 2013 for the only exception in which an adult model was considered). This is the first study in which different inhibition tasks were used to investigate two inhibition components with reference to a model of inhibition tested in children (Gandolfi et al., 2014). Specifically, in the current study, we refer to response inhibition as the ability to control a predominant response and suppressing interference as the ability to respond to one task attribute and to inhibit the response to another attribute. Our results show that individuals with DS show a delay in both of the evaluated inhibition components. Given the importance of inhibition for other cognitive abilities (i.e., working memory, see Lustig et al., 2001; intelligence, see Lee et al., 2015), this evidence suggests that both the ability to control a response and the ability to manage interference must be supported in individuals with DS. More generally, we argue that investigating inhibition in individuals with DS is preferable to using diverse inhibition tasks to achieve information on diverse inhibition components. As suggested by Morra et al. (2017), it is important to pay attention to the way that inhibition tasks are classified based on theoretical assumptions.

## Limitations and future directions

There were some weaknesses in the current study that should be noted. First, although this study aimed to focus on inhibition, it would have been useful to control for other non-executive or executive abilities, such as working memory. Second, although the formative indices may represent a useful methodology to investigate executive functions, in this study, after testing the inhibition model on typical-developmental children with an EFA, we assumed that the inhibition construct was similar in both typical and atypical development. Increasing the sample size would be useful to examine findings observed using reflective and formative inhibition indices (Willoughby et al., 2015) in individuals with DS. Third, the DS group was matched for mental age to the typically developing children. Nevertheless, the DS group showed high variability in chronological age. Consequently, high variability in environmental factors that may have affected inhibition development must be considered. For example, when a large age range is considered, it could be useful to add information concerning the type of treatment and support received and as well as information on differences in treatment that may depend on the cohort to which the subject belongs. To minimize the effect of confounding factors, in future research, it would be useful to consider DS samples with reduced chronological and mental age ranges or to include chronological age-matched TD comparison groups (Godfrey and Lee, 2018).

## Ethics statement

This study was carried out in accordance with the recommendations of the Ethical Code of Italian Psychology Order and of the Ethical guidelines of the Italian Association of Psychology with written informed consent from all subjects. All parents of the subjects gave written informed consent in accordance with the Declaration of Helsinki. At the time we collected the data no ethical committee was yet present to which we could refer to.

## Author contributions

LT and MU: revised the literature on inhibition development; MF and MP: revised the literature concerning inhibition in individuals with Down Syndrome; MU, MP, LT, and MF: conceived and designed the experiment; LT and MF: collected the data; LT and MU: performed the analysis; LT: wrote a first draft of the manuscript that was revised by MF, MU, and MP. All authors read and approved the final manuscript.

### Conflict of interest statement

The authors declare that the research was conducted in the absence of any commercial or financial relationships that could be construed as a potential conflict of interest.
